# Zinc oxide nanoparticles (ZnO NPs) combined with cisplatin and gemcitabine inhibits tumor activity of NSCLC cells

**DOI:** 10.18632/aging.104187

**Published:** 2020-11-20

**Authors:** Chenping Hu, Wenxiu Du

**Affiliations:** 1 Scientific Research Department, Cangzhou Central Hospital, Cangzhou, Hebei Province, China; 2 Respiratory Ward One, Cangzhou Central Hospital, Cangzhou, Hebei Province, China

**Keywords:** nanoparticle, zinc oxide, cisplatin, gemcitabine, non-small cell lung cancer

## Abstract

Non-small cell lung cancer (NSCLC) is one of the most common malignancies worldwide. The use of a combination of chemotherapy drugs and zinc oxide nanoparticles (ZnO-NPs), which have proven to induce tumor-selective cell death, reduce the drug resistance and reduce the side effects *in vitro*. In the present study, we developed ZnO-NPs loaded with both cisplatin (Cp) and gemcitabine (Gem) (ZnO-NPs(Cp/Gem)), then the morphologies and the size distribution of ZnO-NPs(Cp/Gem) particles were observed by transmission electron microscopy (TEM) and dynamic light scattering (DLS). Also, MTT, western blot and Annexin V-PI were used to assess the anti-tumor role of ZnO-NPs(Cp/Gem) in A549 cells. The viability for A549 cells showed a significant decrease in the ZnO NPs(Cp/Gem) group, respectively relative to Cp, Gem, the combination of Cp and Gem (Cp+Gem), and ZnO-NPs loaded with Cp (ZnO-NPs(Cp)) or Gem (ZnO-NPs(Gem)). Furthermore, ZnO-NPs(Cp/Gem) remarkably enhanced the apoptosis-promoting effect of Cp and Gem in A549 cells. The xenograft model showed that Zno-NPS (Cp/Gem) significantly enhanced the inhibition of Cp and Gem on tumor formation. The above results suggested that therapy of NSCLC with ZnO-NPs(Cp/Gem) could enhance the cytotoxic action of chemotherapeutic agents synergistically, indicating a promising potential for ZnO-NPs in antitumor applications.

## INTRODUCTION

Tumors are a group of autologous cells that lose their normal growth regulation mechanism and undergo malignant transformation [1]. They have a high incidence in the population. There are millions of patients who die of malignant tumors every year in the world [2]. People put forward a variety of treatments including surgery, radiotherapy, chemotherapy, and immunotherapy, of which chemotherapy is still the primary treatment. However, chemotherapy is also limited by several factors such as low selectivity, large adverse reactions, and drug resistance [3, 4]. To avoid the above shortcomings, reasonable use of the combination of drugs has more potential for the treatment of tumors [5]. However, decisions about which drugs to use and how to mix doses are often made through experience [5]. Since the efficacy of anti-tumor drugs varies greatly, different tissue types of tumors do not respond consistently to the same drug [6]. Therefore, before the sensitivity test, the quantitative analysis of synergistic, additive and antagonistic effects among anticancer drugs with the principle of median-effect will provide a valuable reference for the correct deployment of anticancer drugs combined chemotherapy in clinical practice [7].

Cisplatin (Cp) and gemcitabine (Gem), as two broad-spectrum anticancer drugs [8], have definite efficacy and high anticancer activity for lung cancer [9]. Unfortunately, Cp and Gem have strong toxic and side effects, so some patients with poor constitution cannot tolerate a complete chemotherapy course, which affects their survival time [10]. Nano-drug carrier technology is a crucial nano-biological technology developed in recent years, which may bring great changes to the treatment of malignant tumors [11]. Currently, nanoparticles have been reported to be promising drug delivery systems, and widely applied into cancer therapy [12–15]. As a new nanomaterial, zinc oxide nanoparticles (ZnO NPs) are a multi-functional inorganic material with broad application prospects [16]. Due to the fineness of particle size, the specific surface area and molecular arrangement, and electronic structure, ZnO NPs possess unique properties such as controllable surface and particle size, excellent stability, and favorable biocompatibility [17]. Besides, the low toxicity and low cost of ZnO NPs also contribute to its wide application [17].

At present, the research on the tumor intervention of ZnO NPs mainly focuses on animal experiments and *in vitro* cell experiments [18, 19]. There are few reports on the animal experiments and cytological studies of ZnO NPs mediating Cp and Gem on lung cancer. In this study, ZnO-NPs were used as drug carriers to carry both Cp and Gem (ZnO-NPs(Cp/Gem)), and then the effects of ZnO-NPs(Cp/Gem) on cell sensitivity, apoptosis and mitochondrial function were investigated in lung cancer A549 cells, which may contribute to understanding the effect and application value of ZnO-NPs(Cp/Gem) in lung cancer chemotherapy.

## RESULTS

### Characterization of ZnO NPs(Cp/Gem)

**Figure 1:**
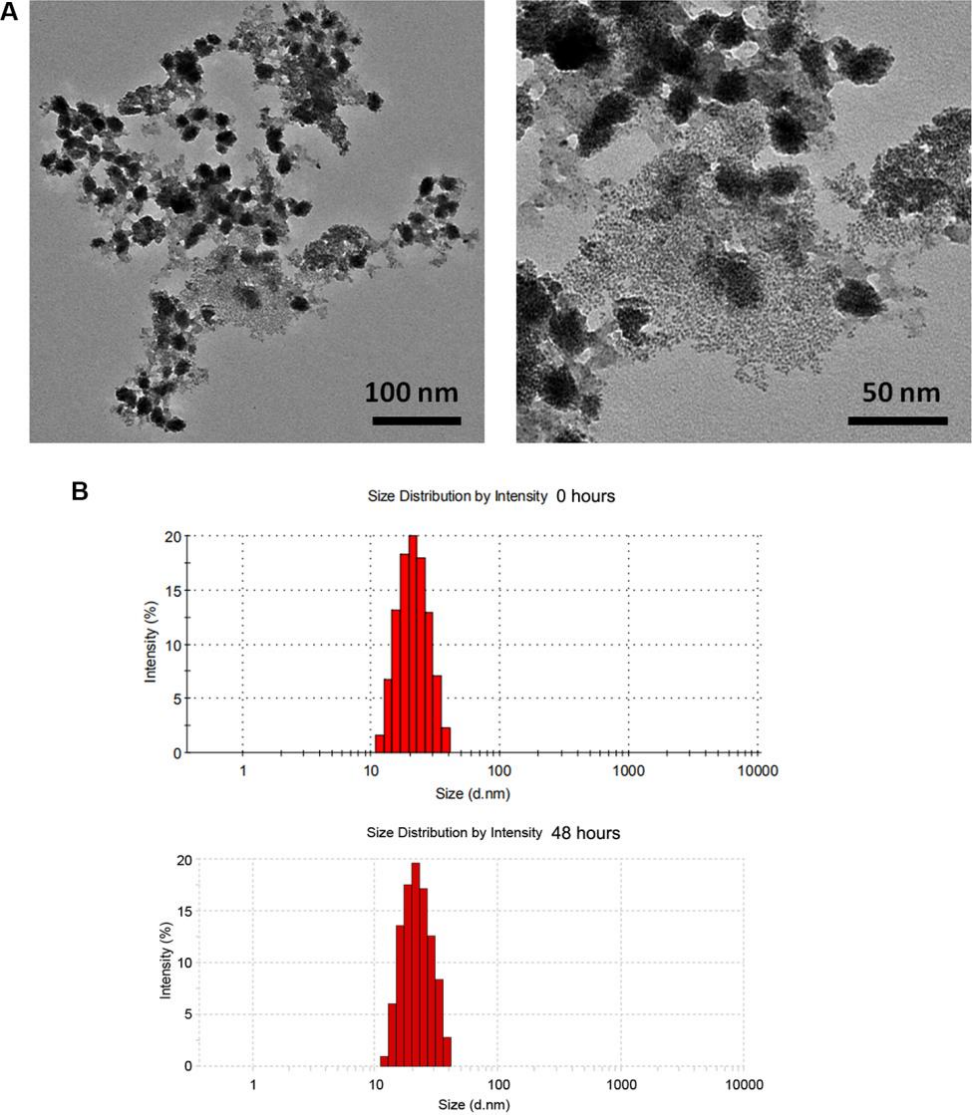
**Physicochemical property of ZnO NPs(Cp/Gem).** (**A**) The morphologies of ZnO NPs(Cp/Gem) by transmission electron microscopy. (**B**) Size distributions of ZnO NPs(Cp/Gem) determined by dynamic light scattering at 0 and 48 h.

As shown in Figure 1A, TEM showed spherical shape of ZnO NPs(Cp/Gem) with the size of ~20 nm. Accordingly, DLS results also showed that the diameter of the ZnO NPs(Cp/Gem) was 21 ± 0.4 nm (Figure 1B). In addition, the DLS results of ZnO NPs(Cp/Gem) were similar between 0 and 48 h (Figure 1B), which suggested the stability of ZnO NPs(Cp/Gem).

### Cytotoxicity of ZnO NPs(Cp/Gem)

**Figure 2:**
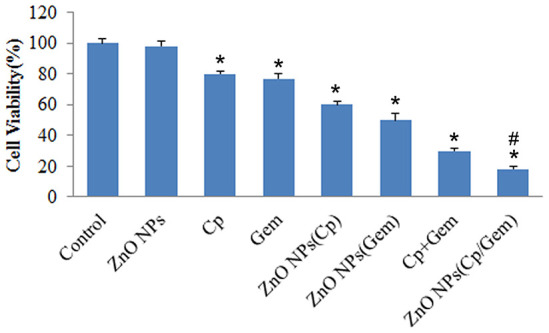
**The toxicity of Cp, Gem, Cp+Gem, ZnO-NPs(Cp), ZnO-NPs(Gem) and ZnO NPs(Cp/Gem) on A549 cells is tested by MTT assay.** * *P*< 0.05 vs. Control. # *P*< 0.05 vs. Cp, Gem, Cp+Gem, ZnO-NPs(Cp), or ZnO-NPs(Gem) groups.

After 24 hours of treatment, the cell activity of the Control group and ZnO NPs group were comparable. However, compared to Control, the viability for A549 cells in Cp, Gem, Cp+Gem, ZnO-NPs(Cp), ZnO-NPs(Gem) and ZnO NPs(Cp/Gem) were all downregulated (Figure 2, *P* < 0.05). Furthermore, the viability for A549 cells showed a significant decrease in the ZnO NPs(Cp/Gem) group, respectively relative to Cp, Gem, Cp+Gem, ZnO-NPs(Cp), or ZnO-NPs(Gem) (Figure 2, *P* < 0.05).

### Cell apoptosis after ZnO NPs(Cp/Gem) treatment

**Figure 3:**
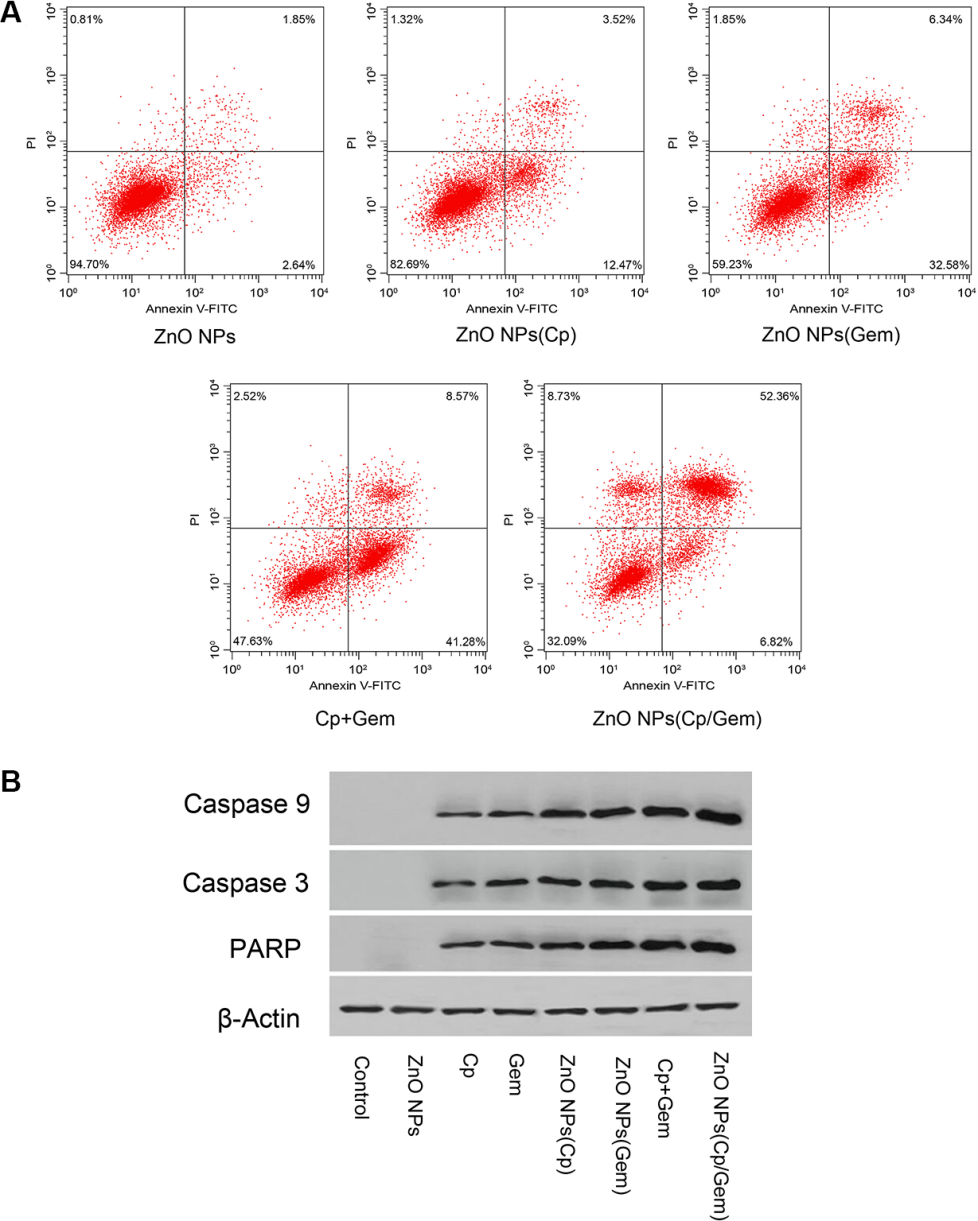
**Effect of ZnO NPs(Cp/Gem) on A549 apoptosis.** (**A**) Apoptosis is tested by Annexin V-FITC/PI method. (**B**) The protein levels of Caspase3, Caspase 9, and RARP is measured by Western blot.

After 24 hours of treatment, partial apoptosis occurred in the ZnO NPs(Cp) group and ZnO NPs(Gem) group, which was higher than that in the ZnO NPs group (Figure 3A). Furthermore, significant apoptosis occurred in Cp+Gem group, which was higher than ZnO NPs(Cp) and ZnO NPs(Gem) groups (Figure 3A). Relative to Cp+Gem, ZnO NPs(Cp/Gem) remarkably upregulated the cell apoptosis(Figure 3A). Furthermore, the expression of pro-apoptotic proteins Caspase3, Caspase 9, and RARP was increased in ZnO NPs(Cp/Gem) group (Figure 3B). The result indicated that ZnO-NPs(Cp/Gem) remarkably enhanced the apoptosis-promoting effect of Cp and Gem in A549 cells.

### Cell ROS level changes after ZnO NPs(Cp/Gem) treatment

**Figure 4:**
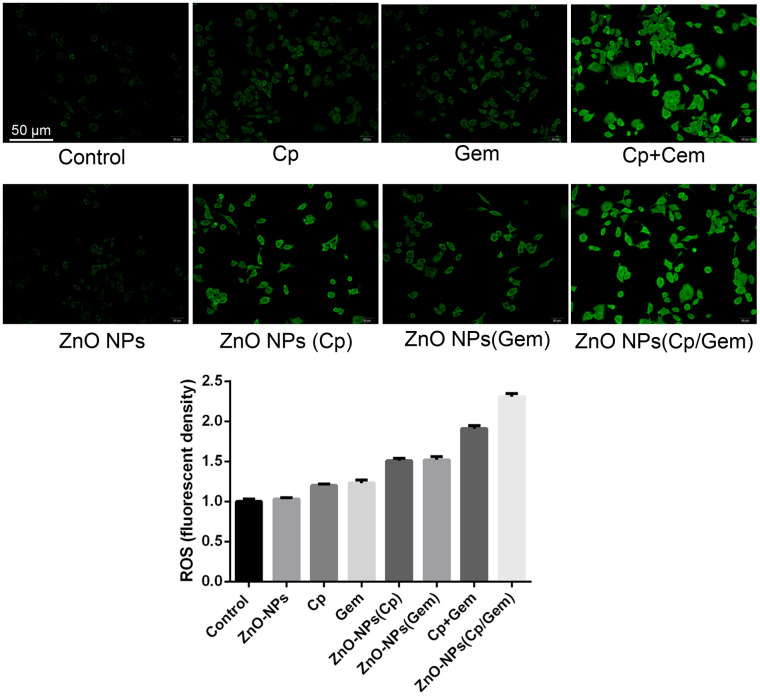
**The ROS levels in the ZnO-NPs, Cp, Gem, Cp+Gem, ZnO-NPs(Cp), ZnO-NPs(Gem) and ZnO NPs(Cp/Gem) groups are tested by CellROX probe method.**

The levels of cellular ROS were tested. The results found that in the Cp, Gem, ZnO-NPs(Cp), ZnO-NPs(Gem), Cp+Gem, and ZnO-NPs(Cp/Gem) groups, the ROS levels in A549 were increased successively, relative to the control and ZnO-NPs groups (Figure 4).

Concretely, few ROS level both in the control and ZnO-NPs groups, while ROS level was increased in the Cp and Gem groups. Notably, compared with Cp and Gem groups, ROS level was significantly increased in the ZnO-NPs(Cp) and ZnO-NPs(Gem) group; meanwhile, ROS level in the ZnO-NPs(Cp/Gem) group was higher than that in the Cp+Gem group (Figure 4).

### Glutathione (GSH) level changes after ZnO NPs(Cp/Gem) treatment

**Figure 5:**
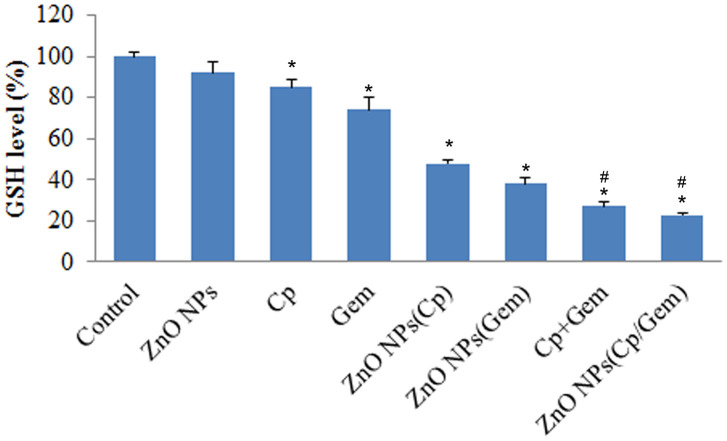
**The GSH levels in ZnO-NPs, Cp, Gem, Cp+Gem, ZnO-NPs(Cp), ZnO-NPs(Gem) and ZnO NPs(Cp/Gem) groups is tested using GSH kit.** * *P*< 0.05 vs. Control. # *P*< 0.05 vs. Cp, Gem, ZnO-NPs(Cp), or ZnO-NPs(Gem) groups.

The GSH levels of the Control group and ZnO NPs group was comparable (Figure 5). However, compared to Control, GSH levels in Cp, Gem, Cp+Gem, ZnO-NPs(Cp), ZnO-NPs(Gem) and ZnO NPs(Cp/Gem) were all downregulated (Figure 5, *P* < 0.05). Furthermore, GSH levels in A549 cells showed a significant decrease in the Cp+Gem and ZnO NPs(Cp/Gem) groups, respectively relative to Cp, Gem, Cp+Gem, ZnO-NPs(Cp), or ZnO-NPs(Gem) (Figure 5, *P* < 0.05).

### ZnO NPs(Cp/Gem) inhibits mitochondrial membrane potential of A549 cells

**Figure 6:**
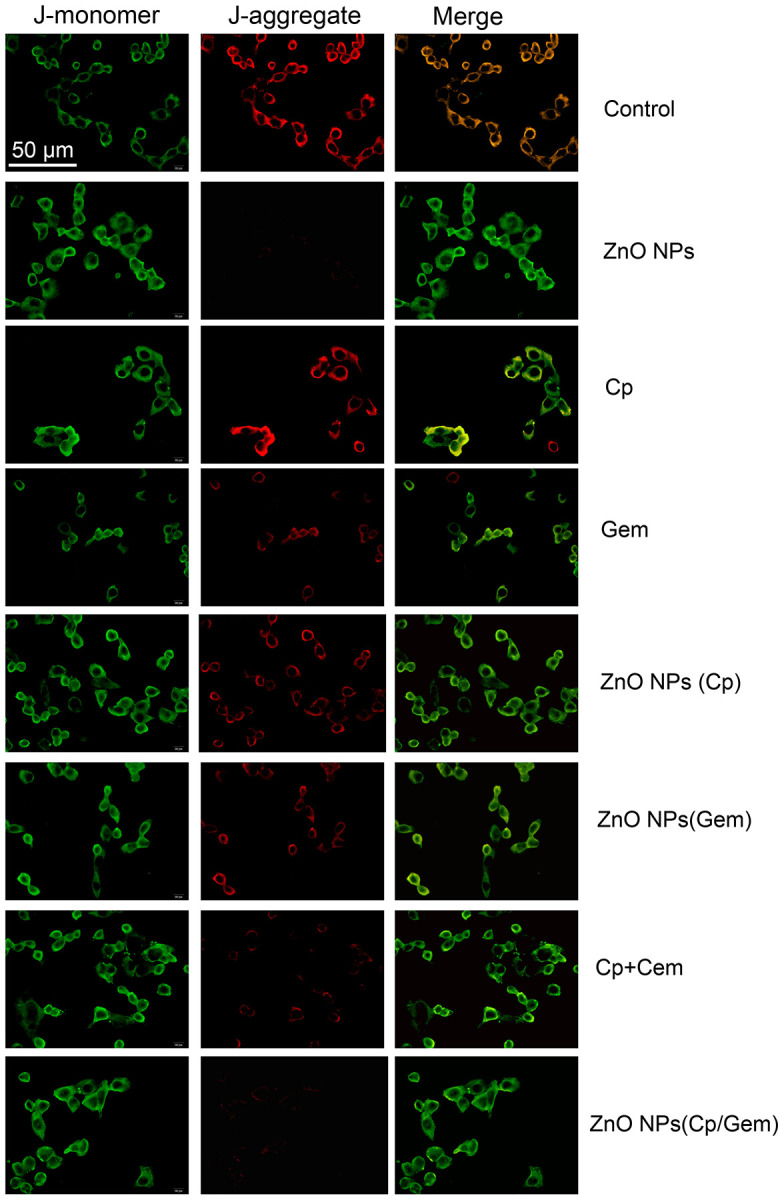
**The mitochondrial membrane potential in A549 cells in ZnO-NPs, Cp, Gem, Cp+Gem, ZnO-NPs(Cp), ZnO-NPs(Gem) and ZnO NPs(Cp/Gem) groups is observed under a fluorescence microscope.**

The mitochondrial membrane potential of A549 cells was tested by flow cytometry. The results showed that A549 mitochondrial membrane potential was downregulated in ZnO NPs(Cp), ZnO NPs(Gem) and ZnO NPs(Cp/Gem) (Figure 6). The mitochondrial membrane potential in ZnO NPs(Cp/Gem) was decreased most remarkably, relative to ZnO NPs(Cp) and ZnO NPs(Gem) (Figure 6).

### In vivo antitumor activity of the ZnO NPs

**Figure 7:**
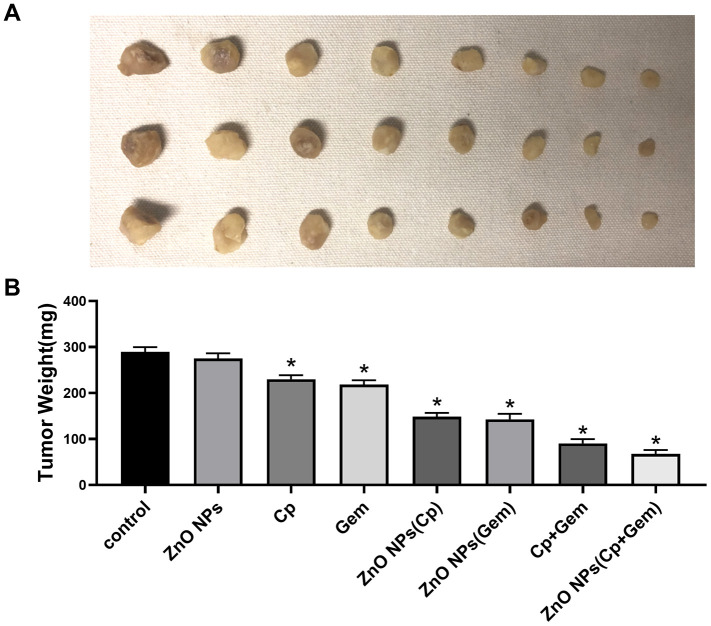
**Anti-tumor activity of ZnO-NPs in vivo.** (**A**) image of subcutaneous xenograft tumors.(**B**) Tumor weight. * *P*< 0.05 vs. Control.

Models of subcutaneous xenograft tumors showed that the tumor weight in ZnO NPs(Cp/Gem) was decreased most remarkably, compared with ZnO NPs(Cp) and ZnO NPs(Gem) (Figure 7).

## DISCUSSION

Human lung adenocarcinoma A549 is widely used as a lung epithelial cell in the *in vitro* study of lung toxicology [20–22]. The cell is easy to culture and sensitive to external factors, so it is more suitable for *in vitro* toxicity of nanomaterial [23]. Targeted improvement of chemotherapy drugs by nanotechnology is a better method to improve the sensitivity of chemotherapy drugs [24]. In the present study, we successfully prepared ZnO-NPs(Cp), ZnO-NPs(Gem) and ZnO NPs(Cp/Gem). Compared with free Cp and Gem, ZnO-NPs(Cp) and ZnO-NPs(Gem) significantly diminished cell viability, inhibited GSH level, increased ROS level, lowered the mitochondrial membrane potential, as well as induced cell apoptosis in A549 cells. Importantly, ZnO NPs(Cp/Gem) exerted the most significant anticancer effects in A549 cells.

In the study of the effect of nano-ZnO on cell growth and metabolism, the MTT method can be used to evaluate the survival rate of cells [22]. It was found that nano-ZnO actively targeted to cancer cells compared with nano-SiO2, TiO2 and other materials [25]. In the present study, we found that ZnO-NPs did not reduce the viability of A549 cells, indicating the low toxicity of ZnO-NPs. Additionally, ZnO-NPs(Cp), ZnO-NPs(Gem) and ZnO NPs(Cp/Gem) were highly toxic to cultured A549 cells at various time points, and cell viability is negatively correlated with ZnO NPs(Cp/Gem) concentration. In addition, GSH is a small molecule peptide which combines with free radicals and heavy metals, to convert the harmful toxins in the body into harmless substances and excreted out of the body. When the damage or permeability increases, resulting in a significant decrease in the activity of GSH in cells. Therefore, the downregulation of GSH activity in cells can reflect the cytotoxicity of drugs [26]. This study found that the variation pattern of GSH levels in ZnO-NPs, Cp, Gem, Cp+Gem, ZnO-NPs(Cp), ZnO-NPs(Gem) and ZnO NPs(Cp/Gem) groups is similar to that of the MTT results, indicating that the toxicity of A549 cells in the ZnO NPs (Cp/Gem) group was significantly higher than other groups. The results suggest that ZnO NPs (Cp/Gem) can cause toxic injury via inhibiting GSH.

Current research shows that reactive oxygen species (ROS) caused by nanoparticles may be one of the main causes of cytotoxicity of nanoparticles [27]. Electron acceptor and donor activity sites on the surface of nanoparticles can act with molecular oxygen (O2) to form superoxide ions and generate excess ROS through disproportionation [28]. Excess ROS can increase the oxidative stress in cells or organisms and attack nucleotides, leading to DNA fragmentation [29].

Furthermore, overproduction of ROS can promote the markers of oxidative damage, including malondialdehyde (MDA) and superoxide dismutase (SOD), catalase (CAT), which resist the damage of superoxide anion and decompose hydrogen peroxide, reducing the oxidative damage of cellular DNA [30, 31]. The results showed that the ROS levels in the ZnO NPs (Cp/Gem) group were significantly higher than those in the control group. This result indicates that ZnO NPs (Cp/Gem) nano-drugs cause excessive ROS production in cells, leading to intracellular oxidation and antioxidant status. Loss of balance, oxidative stress reaction [32, 33]. It is confirmed that the reactive oxygen species caused by ZnO NPs (Cp/Gem) nano drug-loaded particles may be one of the main reasons for the cytotoxicity of nanoparticles.

Apoptosis is an active form of death after exogenous or endogenous stimulation of cells [34]. Rahman et al. [35] applied nano-carrier particles with a particle size of less than 20 nm to primary rat embryonic fibroblasts, and the number of micronuclei increased significantly, and cell membrane blisters and apoptosis bodies are formed, and a ladder-like random rupture of DNA has been observed. Additionally, after treatment with ZnO NPs on neuroblastoma cells, the chromosomal chromatin aggregation can also be found by TEM, indicating that the nano-carrier particles induce apoptosis [36]. It was demonstrated that the oxidative stress generated by the highly reactive nanoparticles causes the rupture of the lipid layer of the cell membrane, and the intracellular calcium homeostasis is out of balance, resulting in the activation of the endonuclease dependent on the concentration of Ca^2+^ ions, causing the cell to wither [37]. Consistent with previous studies, our results observed that ZnO NPs (Cp/Gem) caused the upregulation of the number of apoptotic cells, and induced apoptosis in A549 cells cultured *in vitro*. Combined with cellular oxidation index and cellular reactive oxygen species detection, we believe that the oxidative stress produced by ZnO NPs (Cp/Gem) is closely related to the apoptosis of A549 cells.

Due to poor water solubility, many hydrophobic anticancer drugs are clinically limited to apply into cancer treatment. The ZnO-NPs have been reported to be able to overcome the application limitation of hydrophobic drugs because of its attractive properties, including controllable surface and particle size, good stability, and favourable biocompatibility, low toxicity and low cost. Importantly, at physiological environment, ZnO NPs are insoluble, while ZnO can directly degrade to non-toxic zinc ions at acidic environment [38, 39]. It is well-known that the lysosome and late endosome usually exist in tumor cells, which results in the acidic tumor microenvironment, thereby ZnO NPs may preferentially target tumor cells [38, 39]. In addition, NPs have the enhanced permeation and retention effect on cancer cells due to aggregation of nanoparticles in tumor tissues [12, 13]. Herein, Cp and Gem as a hydrophobic anticancer drug were loaded into the ZnO-NPs in order to improve drug deliver. This study revealed that ZnO-NPs(Cp), ZnO-NPs(Gem), and ZnO NPs(Cp/Gem) exerted the better anticancer effect, especially ZnO NPs(Cp/Gem), in compared with free Cp, Gem and Cp+Gem, which suggested that ZnO-NPs improved the long-term chemotherapy efficacy for cancer cells.

In this study, we found that ZnO-NPs loaded with both cisplatin (Cp) and gemcitabine (Gem) (ZnO-NPs(Cp/Gem)) were prepared successfully, which increased the toxicity of Cp and Gem and reduced apoptosis of NSCLC cell line A549. More importantly, the glutathione levels and mitochondrial membrane potential of A549 were downregulated after treatment with ZnO-NPs(Cp/Gem). It is indicated that ZnO-NPs(Cp/Gem) is a highly promising nano drug-loading system. In conclusion, ZnO NPs (Cp/Gem) exerted significant anti-tumor effect by reducing the growth activity of A549 cells and destroying the integrity of the cell membrane.

## MATERIALS AND METHODS

### Preparation of ZnO-NPs(Cp/Gem)

ZnO-NPs (particle size 20 nm) were purchased from Jiangsu Haitai Nanomaterial (Jiangsu, China). To prepare ZnO-NPs(Cp/Gem), Cp and Gem (both obtained from Sigma-Aldrich, St Louis, MO) were respectively dissolved in 1 ml of anhydrous methanol, and triethylamine (the mass ratio of Cp or Gem to triethylamine is 1:2) was added to obtain hydrophobic Cp or Gem. Then, the above solutions were mixed with 3 ml ZnO-NPs solution dissolved in chloroform (the ratio of the ZnO-NPs to Cp and Gem was 1:5: 5), the mixed solution of Cp, Gem, and ZnO-NPs was transferred to an eggplant bottle. Organic solvents were removed by vacuum rotary evaporator to form a dry ZnO-NPs(Cp/Gem) at the bottom of the bottle.

The morphologies and the size distribution of ZnO-NPs(Cp/Gem) particles were observed by transmission electron microscopy (TEM) and dynamic light scattering (DLS), respectively. Briefly, ZnO-NPs(Cp/Gem) solution was diluted with deionized water at 2 uM, and then 10 μL of the sample was dropped onto the carbon coated copper mesh. After water evaporation, the sample was counterstained with 5 μL of 1% uranyl acetate solution for 30 s, and dried by a 42° C constant temperature dryer. Ultimately, the sample was observed by TEM (Tecnai G2 20 S-TWIN, FEI, Eindhoven, Netherlands). DLS was performed by Zetasizer Nano Z (Worcestershire, UK) at 0 and 48 h, respectively.

### Cell culture

Human lung cancer A549 cells were obtained from American Type Culture Collection (ATCC). The cells were cultured in RPMI-1640 medium containing 10% BSA, 100 U/ml penicillin and 100 U/ml streptomycin with pH value of 7.2 - 7.4 in 37° C with 5% CO_2_ in a humidity incubator. In this study, the cells were grouped as Control, ZnO NPs, Cp, Gem, combination of Cp and Gem (Cp+Gem), ZnO-NPs loaded with Cp (ZnO-NPs(Cp)) or Gem (ZnO-NPs(Gem)), and ZnO-NPs(Cp/Gem).

### Cell viability by MTT

A549 cells in logarithmic growth phase were collected by 0.25% trypsin digestion. The cell suspension was prepared in DMEM medium, adjusted to a cell concentration of 5×10^4^ mL, seeded in a 96-well cell culture plate (100 μL/well), and cultured at 37° C for 12 h. The control group was replaced with 100 μL of cell culture medium containing 0.025% sodium carboxymethylcellulose, and the experimental group was replaced with 100 μL of the above five concentrations of nano ZnO particle suspension. After 12 hours of incubation, MTT solution (5g/L) was added at 10 μL/well. After incubating for 4 h, the supernatant was removed, 150 μL of DMSO was added to each well, and the cells were gently shaken for 30 s. 100 μL of each well was aspirated into the enzyme label, and then the absorbance value (OD) at 570 nm was measured by a microplate reader. The same method was used to detect co-culture with ZnO at 24 h, 48 h, and 72 h. The percentage of cell activity was used as an evaluation index for the effect of nanoparticles on cell viability. The calculation formula was: percentage of cell activity (%) = (OD value of each well in the infected group - blank OD value) / (Control group OD mean - blank OD value) × 100%, control group cell activity percentage was 100%.

### Reactive oxygen species (ROS) production in A549 cells

The level of intracellular ROS was measured by a fluorescent probe method. The CellROX™ Deep Red Reagent (C10422, Thermo Fisher Scientific, Thermo Scientific ™, Waltham, MA, USA) was diluted with a serum-free cell culture medium at a ratio of 1:1000 to remove the cell culture medium in the six-well plate, each well added 1 ml of diluted CellROX to the well, and was incubated at 37° C for 30 min in dark, then the supernatant was discarded. Then the cells were washed with serum-free cell culture solution 3 times to completely remove CellROX that had not entered the cells. After that, the cells were irradiated with 120 mg/cm^2^ UVB for 120 s and were directly observed with a fluorescence microscope.

### ROS production in mitochondria

Detection was performed using a CellROX probe (C10422, Thermo Fisher Scientific). 50 μg of CellROX was dissolved in 13 μL of DMSO-prepared 5 mmol/L CellROX dye solution to prepare a 5 μmol/L Mito SOXTM working solution. After the cell intervention was terminated, the cells in the 6-well plate were washed twice with D-Hank solution; 1 μL of 5 μmol/L CellROX working solution was added, and incubation was carried out for 10 min at 37° C in the dark; D-Hank solution was lightly washed 3 times, each well. 1 mL of D-Hank solution was added and observed under an inverted fluorescence microscope and photographed. Image semi-quantitative analysis by Image-J software was used to calculate the average fluorescence intensity per unit cell area.

### Changes of mitochondrial membrane potential

Cells in each group with 10^-5^ mol/L was added with 0.5 ml of freshly prepared JC-1 working solution and were incubated at 37° C for 4 h, 12 h, and 24h. Then the cells were washed with 2 ml and 1 mL 1× analysis solution. The cells were suspended in 0.5 mL of 1× analysis solution and were observed under a fluorescence microscope.

### Cell apoptosis assay

Resuspend the cells in the logarithmic growth phase of each group, take 50,000-100,000 cells, centrifuge at 1000 g for 5min, discard the supernatant and add 195 ml Annexin V-FITC binding solution to resuspend the cells, and stain according to the instructions of the apoptosis detection kit. Apoptosis was detected by a post-flow cytometer. The results consisted of four quadrants, including normal cells [An(-)/PI(-), at the lower left quadrant], early apoptotic cells [An(+)/PI(-), at the lower right], late apoptosis and necrosis cells [An(+)/PI(-), at the upper right], and then the damaged cells ([An(-)/PI(+), at the upper left], and the apoptosis rate was calculated as follows: the ratio of early apoptosis and the proportion of late apoptotic cells to the total cells.

### Western blot

Each group of the cells was washed 1-3 times with pre-cooled PBS, and RIPA lysate was added to an ice bath for 30 min, and the cells were shaken once every 10 min. The cells were scraped off with a cell scraper and transferred to a 1.5 ml EP tube. The protein was separated by centrifugation at 12000 r/min for 30 min at 4° C. The supernatant was removed and the protein was quantified using a BCA protein quantification kit. Each 100 mg of protein was added 1:1 to 1×SDS loading buffer and the protein was boiled for 10 min to denaturation. After 12% SDS-PAGE electrophoresis, the above proteins were transferred to a PVDF membrane after electrophoresis, and the Caspase3, Caspase9, PARP protein bands were detected after antibody incubation.

### Animals

Animal experiments were permitted by the Animal Protection and Ethics Committee of Cangzhou Central Hospital. BALB/c nude mice were purchased from Beijing Weitong Lihua Experimental Animal Technology Co., Ltd. (Beijing, China). For the experiment of Xenograft, A549 cells (5 × 106) were suspended in 200 μl normal saline and injected subcutaneously. Intraperitoneal injection of the drug was given once every other day.

### Statistical analysis

GraphPad Prism 7.0 was used for statistical analysis of experimental data. One-way ANOVA and Student's t-test were used for comparative analysis of differences. *P*< 0.05 was considered statistically significant.
